# Musca Domestica in Tuberculosis

**Published:** 1908-06

**Authors:** Theo. Y. Hull

**Affiliations:** San Antonio, Texas


### Musca Domestica in Tuberculosis.

BY THEO. Y. HULL, B. S., M. D., SAN ANTONIO, TEXAS.

In one of the larger cities a few years since the people were considerably agitated over the discovery that danger lurked in the bright red apples, temptingly displayed to attract the school children by the curbstone merchants. It was found that one of these who had daily purchased fruit from a push-cart vendor had contracted a much-dreaded malady. The origin of the infection, on investigation, was clearly proven. I have seen negro hucksters taking fruit from a room in which a child lay dangerously ill of diphtheria to dispose of it upon the streets. We are most of us familiar with the nauseating practices of the "banana man," who, for want of a better place, deposits the unripened fruit in the putrified atmosphere of his bedchamber to mature and mellow. But it remained for one of the great markets in our Capital City—one of the most sanitary cities in the world—to most forcibly present to my mind the potency of the common house fly—musca domestica—as an agent in the dissemination of bacteria pathogenic to man.

On this particular occasion I had entered one of the large markets for the purpose of ordering the daily meat supply, etc. These markets are usually kept in a reasonable state of cleanliness but not always strictly sanitary. As I approached my point of destination I noticed the proprietor of a neighboring stand coughing violently and expectorating freely. At the same time I caught sight of quantities of fresh meat exposed for sale covered with flies. It immediately occurred to me that I had no desire for meat that day, and I practiced strict vegetarianism for many hours thereafter. The possibility of particles of sputum being transported from one stand to the other was too great. At that time the general public was not so enlightened on the subject of bacteriology and the possibility of food infection in that way as in these days of war upon all kinds of micro-organisms.

It is not altogether impossible now to see large quantities of meat and other articles of food, raw and cooked, in the markets and on the restaurant counters exposed to the possibility of contamination through this same agency. Many of our cheap eating houses are absolutely filthy in this respect. The markets are not fly proof, neither are the meat blocks aseptic, notwithstanding the reported use of antiseptics in cold storage plants. The exposure of food materials to the immediate inspection of the would-be purchaser seems a necessity, yet the distance from the open street, where all may and many do expectorate freely, the refuse box and the sawdust covered floor does not preclude the danger of contagion being imparted in this way. A little reflection along this line while not altogether cheerful and appetizing may be profitable. It may cause us to think of some things which, because common, are not thought to be sources of evil. It may also cause us to wish that we might breathe filtered air, drink distilled water and certified milk and eat superheated food kept under aseptic conditions and served on sterilized dishes.

All are familiar with the crusades against the mosquito, another member of the same order, diptera, and all are agreed, save a few doubting Thomases, as to the necessity of limiting its field of action. The discovery of the anopheles as the active agent in malaria, the stegomyia fasciata in yellow fever and the high probability that dengue is due to a similar species, has stimulated the study of these and like agents as the source of infectious diseases. The knowledge that the stegomyia acts as a perfect incubator and after the twelfth day is capable of distributing its culture products for nearly two months to all susceptible callers, has led to the belief that other insects of the same order may in a like manner be prolific dispensers of evil.

A similarity in the gross anatomy of the musca domestica, its lowly origin, and its disgusting habits lends probability to its being a potent factor in the distribution of disease germs. If one will take the trouble to place the head of a common house fly under the microscope, maynifying it not to exceed 105 times, and examine the mouth organs with their hairy appendages, he will be struck by the perfect adaptability of these parts to gathering up and retaining particles of sputum and other germ-laden matter. The hairy legs and feet show the same perfect adaptation to purpose, and many particles of matter may be seen hanging to them. Now, if one takes a small particle of the sputum, not larger than a pin head, from some case of consumption, and prepares it in the usual manner to bring out the bacilli and cocci, he may learn another striking lesson. This small particle of sputum when properly spread on a cover glass will extend over an area one-third inch in diameter. Placing the preparation under the microscope and using an oil immersion lense capable of magnifying one thousand diameters attempt to count the tubercle bacilli and other pathogenic bacteria. I have prepared slides in which the tubercle bacilli were more numerous than the traditional "hairs of the head," simply countless, and streptococci and staphylococci and influenza bacilli a hundred to one. This is a "mixed infection in which the patient often expectorates several ounces daily. The particle examined was only of pin-head dimension. Remember the myriads of flies everywhere.

Just what takes place in the alimentary canal of the fly we do not definitely know, but this we do know, that the digestive ferments of that canal are not strong enough to destroy many species of pathogenic bacteria. Whether there is or is not an actual increase in their numbers during the interval between ingestion and egression is not positively known. That the dejecta of flies contain numerous virulent bacteria has been proven beyond dispute. This fact is all important. It is our main contention.

As to the habits of the house fly, it is largely a scavenger. Wherever there is filth or offal or decomposing matter it seeks its food and deposits its eggs. The filth of the barnyard, the most nauseating deposits of the street and the choicest food of the table are each tempting items on its daily menu. The musca, like many other members of the numerous order of diptera, have a great fondness for sweets, but do not disdain to help themselves to the oatmeal, the cream cheese, the cold meat, the pitcher of milk or any other dish the board may afford. Its activity is ceaseless. Its appetite is enormous and not at all particular. Its social qualities are of high order; in other words, as most politicians and some physicians understand it, a "good mixer." All day it plies its vocation with tireless energy, now in the stable, now in the sick room, and now taking a sip from the glass of milk you were drinking, or helping itself to the soup and the meat, the pie and the cake you ordered for dinner. Into the cellar and pantry, into the kitchen and dining room, wherever the odor of food attracts it goes and deposits its germ-laden dejecta.

Let us now look at this subject from another standpoint. In all probability a tuberculosis census properly taken would reveal a million cases in the United States. Nearly one-half of this number may be called "consumptives," that is, those who have passed into the later stages of the disease which usually lasts from two to three years, during which time expectoration is free. Statistics show that about 160,000 die annually from this one cause. This is, in all probability, much less than the actual number. Again, the carelessness of invalids suffering from chronic diseases is proverbial. Sometimes it is due to ignorance and sometimes to indifference. But from whatever cause the fact remains that a proper disposition of the sputum, the urine and the feces of consumptives is not made. The average invalid seems to know very little concerning such sanitary matters, and in some districts it is not an uncommon thing to see these thrown upon the ground at no great distance from the house. These invalids are simply untrained and know nothing of the dangers of reinfection to themselves or of infection to others. In not a few instances I have found them ignorant of the true nature of their malady, and of course in such cases we do not expect much care. Some I find swallowing large quantities of sputum daily with the consequent loss of appetite and indigestion. In these cases and in cases of intestinal tuberculosis, the feces will contain large numbers of virulent bacilli. In those cases in which lesions exist in the urinary tract large numbers may also be found in the urine.

Many patients I find provide no proper receptacle for the sputum. An open tin pail or can containing a little water, with sometimes a dash of carbolic acid, but often without, is commonly used when the expectoration is profuse. Others spread a piece of newspaper upon the bed or carpet, particularly at night, to serve as a cuspidor. Bits of old cloths often answer the purpose, sometimes the stove hearth or open grate, and oftener still the open window. It is common to see the sick expectorating in the street and near the steps of the corner grocery store. The markets and groceries seem to attract strongly the ambulatory cases. In over one-half the cases a sanitary spitting cup is unknown, and the use of one is openly resisted by not a few. All this, of course, affords the musca domestica a rare opportunity for feasting.

For many years the medical world has looked upon tuberculosis as a "dust borne disease." It has looked upon the infection of the respiratory tract as direct through the inhalation of the bacilli with the air. To such an extent was this belief held that very little attention was given to any other possible channels of infection. That infection does take place through other channels is now an established fact. The investigations of the last few years have been fruitful, and we have come to know that the stomach acts as a receptacle for vast numbers of bacilli and that very many people are infected in this way. Much of the dust inhaled with the air lodges on the moist walls of the pharynx and is ultimately washed into the stomach. Tuberculosis in children may be due largely to drinking the milk of tuberculous cows, though the identity of human and bovine tuberculosis is somewhat in doubt. Lately it has become evident that some contract the disease through eating diseased meat. A result of this knowledge is seen in the recent and most general agitation for a pure milk supply and to prevent the sale of diseased meat in some cities. Now when we fully realize that we can contract tuberculosis through eating contaminated food we are in a position to see the necessity for some very simple sanitary measures.

As I have said before, the common house fly is a very social creature and a good mixer and not at all inclined to confine his operation to a single room or in fact to a single house. It is possible for it to reinfect a tubercular person and every other member of the family and also the neighbors of such an invalid. Probably very little thought has been given to this danger of infection. My own observations lead me to think that no thought is given to it. The general ignorance of the laity in such matters is lamentable. They have learned the danger of mosquitoes disseminating malaria and yellow fever in many districts, but they have given very little thought to so common a thing as the ordinary house fly and its possibilities for doing evil.

This source of danger of contagion is no illusion. Numerous virulent bacilli have been found in the dejecta of flies. In a few hours after feeding upon tubercular sputum they begin to deposit bacilli everywhere, on the ceiling, in the food and not unfrequently in the mouths and nostrils of sleeping children. For the sake of illustration we will suppose a very common situation to exist. Across the street lives an invalid in the last stages of consumption. The need of fresh air is apparent. The doors and widows are wide open. The house is not screened. The patient uses an open receptacle for the sputum, which is copious. By and by, a neighbor opposite prepares a tempting supper. The aroma of various cooked dishes spreads abroad. In short order musca domestica being entirely free to go where he lists, desires a change of diet. The tempting odors are too tempting to go uninvestigated. He is probably somewhat thirsty and takes a sip of milk here and another there and tries first this dish and that dish while the busy servant is thinking of other things or cares not. It is possible for a few flies in this way to infect a whole family in which no sickness now exists. By and by that happy circle will be broken, for a foe has entered unobserved. Then, when too late, the common cry, "how was it contracted?" They are sure it is not hereditary in the family. The answer is not difficult to find. Oh, thoughtless ones and indifferent ones, think and care just a little! There are other bodies that can suffer and other hearts that may break. Ignorance will not always excuse and indifference is damnable.

How can this danger of infection be prevented? It will not be a very difficult matter. Physicians have it largely in their power to accomplish it. A campaign of education is necessary. It is now going on and school children are being taught things of much importance. The sick of tuberculosis must be made to know the danger in using an open receptacle for the sputum, that danger also lies in the feces and urine, that all of these must be destroyed with proper germicides and that a sanitary spitting cup is an absolute necessity. This should be insisted upon by every physician. It is one of the duties he owes the community. There is absolutely no excuse for sputum being accessible to flies. As a further protection every house should be carefully screened and flies excluded. Again, flies should be exterminated as far as possible. This may seem like attempting the impossible, but much can be done that is not done. It was once thought impossible to exterminate the mosquito, but a little wholesome brushing up and cleaning up has worked wonders. Likewise a few simple sanitary measures may prove a revelation.

A small thing, because unusual, may create a great commotion, while a great thing, because common, produces no commotion whatever. A great city was greatly agitated over a single case of syphilis contracted in an unusual way, but it slept on undisturbed by the fact that a *hundred thousand* cases of tuberculosis existed within its limits. A single case of yellow fever in New Orleans will receive prompt attention from every newspaper in the United States, and the whole machinery of the government will be put in operation to corral it, while the annual death of over two hundred thousand victims of the "white plague" fails to arouse even passing comment. The condition of the public at large in reference to tuberculosis is apathetic, and apathy is not indicative of progress.

Now let us review briefly the local condition. In every city the fly and dust nuisance has a direct relation to the exposure of food materials along the streets and in the markets. If it be true that even a small per cent of the cases of tuberculosis owe their infection to the consumption of infected food, then the question of the sanitary condition of our grocery stores, butcher shops and markets becomes of serious importance, for the eating of infected meat, the drinking of infected milk and the consumption of fruits that have been exposed to the flies and dust of the street may be fraught with serious consequences.

The exposure of food material, especially that which is to be eaten without further cooking, to the possibility of contamination should be prohibited by the most stringent health regulations.

Recently, in order to determine what precautions were taken by dealers in articles of food to preserve the same in a wholesome state and to prevent their contamination from dust and flies, I made a short tour of inspection. In all I noted the condition of some fifty places where apples, oranges, berries, cakes, candies, etc., were exposed for sale. I also noted the condition of the streets and the evidence of expectoration upon the sidewalks. In very few places did I find any attempt to exclude either flies or dust, and most of these were very defective. In over twenty places fruit and berries were exposed to nearly everything that came along, including dogs. In a distance of less than two blocks I counted twenty-five places where persons had recently expectorated upon the sidewalk near the building line. Much of this had the appearance of tubercular sputum. The streets were extremely dusty and the dust was in a constant state of disturbance by the wind, street cars and automobiles.

Now I will lay down this proposition: First, given fruit and berries exposed for sale, and masses of tubercular sputum at no great distance upon the sidewalk; second, innumerable flies and clouds of dust from street cars, automobiles and wind. What will be the result? Will the fruit and berries be free from all danger of contamination?

I asked one groceryman if he had any berries that were not exposed. He replied, "No," and added that I could wash them. He might have also added that I could cook them. The fact, however, remains that most berries are eaten raw and many do not even wash them, and they would not be apt to wash off particles of tubercular sputum if they did. This material dried upon a smooth surface can scarcely be removed with any amount of washing.

Now let us view this from another point of view. We live in a city in which the death rate from tuberculosis is the second highest in the United States (567.7 per 100,000, Cen. Rep., 1906), and her nearest rival for third place is nearly 40 per cent lower.

With this number of sick people in our midst, a warm climate, and not the best of sanitation, what is the danger to ourselves and our children? The same question may be asked in nearly every city in Southwest Texas.

The public looks to the medical profession for advice in sanitary matters, even though it does not follow it. What shall our advice be in matters of this kind?

---

LUMBAGO.—A good remedy for all forms of lumbago is:

℞ Specific arnica
Specific macrotys, aa........................ʒij

M. Sig. Dose, two to four drops every one or two hours.—*Burnett, Wisconsin Med. Recorder.*

